# Tubulin Binds to the Cytoplasmic Loop of TRESK Background K^+^ Channel *In Vitro*

**DOI:** 10.1371/journal.pone.0097854

**Published:** 2014-05-15

**Authors:** Péter Enyedi, Irén Veres, Gabriella Braun, Gábor Czirják

**Affiliations:** Department of Physiology, Semmelweis University, Budapest, Hungary; University of South Florida College of Medicine, United States of America

## Abstract

The cytoplasmic loop between the second and third transmembrane segments is pivotal in the regulation of TRESK (TWIK-related spinal cord K^+^ channel, K2P18.1, KCNK18). Calcineurin binds to this region and activates the channel by dephosphorylation in response to the calcium signal. Phosphorylation-dependent anchorage of 14-3-3 adaptor protein also modulates TRESK at this location. In the present study, we identified molecular interacting partners of the intracellular loop. By an affinity chromatography approach using the cytoplasmic loop as bait, we have verified the specific association of calcineurin and 14-3-3 to the channel. In addition to these known interacting proteins, we observed substantial binding of tubulin to the intracellular loop. Successive truncation of the polypeptide and pull-down experiments from mouse brain cytosol narrowed down the region sufficient for the binding of tubulin to a 16 amino acid sequence: LVLGRLSYSIISNLDE. The first six residues of this sequence are similar to the previously reported tubulin-binding region of P2X2 purinergic receptor. The tubulin-binding site of TRESK is located close to the protein kinase A (PKA)-dependent 14-3-3-docking motif of the channel. We provide experimental evidence suggesting that 14-3-3 competes with tubulin for the binding to the cytoplasmic loop of TRESK. It is intriguing that the 16 amino acid tubulin-binding sequence includes the serines, which were previously shown to be phosphorylated by microtubule-affinity regulating kinases (MARK kinases) and contribute to channel inhibition. Although tubulin binds to TRESK *in vitro*, it remains to be established whether the two proteins also interact in the living cell.

## Introduction

Two-pore domain (K2P) K^+^ channels are the widespread molecular correlates of background (leak) potassium currents in native cells [1]. Some members of this family (e.g. TASK and TREK channels) stabilize the negative resting membrane potential and counteract depolarization in a great variety of cell types both in the central nervous system [2]–[4] and in peripheral tissues [5]–[9]. Some other K2P channels, however, are distinguished by a more circumscribed expression pattern. TRESK has originally been described as a potassium channel of the human spinal cord [10], afterwards it was also found in mouse cerebellum [11], testis, thymus and spleen [12]. Later, general consensus was reached that TRESK is robustly expressed in pseudounipolar neurons of dorsal root, trigeminal and other sensory ganglia [13]–[19]. At present, these ganglia are considered as the major location of the channel, although some evidence is accumulating that it may also be important in ganglia of the autonomic nervous system [20], and in lymphoblastic cell lines [21], [22].

TRESK of the trigeminal ganglion has been implicated in migraine pathogenesis [18], [23]–[26], and accordingly, there is an ongoing effort to develop TRESK openers for therapeutic intervention in this frequent neurological disorder [27]. In this way it is possible to take advantage of what has been learned about the channel, although there are still significant gaps in our understanding of TRESK function and regulation in native cells. Methods for the selective measurement of TRESK current in sensory neurons have not yet been developed, and the plasma membrane regions containing high density of the channel have not been unequivocally established in this elaborate cell type. Thus the investigation of TRESK regulation in dorsal root ganglion neurons is still elusive.

The majority of our knowledge about the general features of regulation of the channel comes from heterologous expression systems. TRESK is regulated in a unique manner; it is activated by the calcium signal in contrast to the other members of the K2P channel family. The activation is mediated by the calcium/calmodulin-dependent protein phosphatase calcineurin [11]. Calcineurin binds to a six amino acid docking motif (a Nuclear Factor of Activated T cells (NFAT)-like site) in the intracellular loop of TRESK and removes inhibitory phosphorylation in response to the elevation of cytoplasmic Ca^2+^ concentration [28]. One regulatory serine (RSNSCPE) in the cytoplasmic loop of TRESK is phosphorylated by protein kinase A [29], and thereby it allows the docking of 14-3-3 adaptor protein to this mode 1 binding site [30]. Another cluster of inhibitory serines (also located in the intracellular loop, RLSYSIISNL) may be phosphorylated by microtubule-affinity regulating kinases (MARK kinases), but not by several other kinase types [31]. We [1] and others [32] found that the Ca^2+^-dependent regulation is operational in the human and mouse channels but not in the distantly related zebrafish TRESK (GQ304739, KC577586), which does not contain the NFAT-like calcineurin-binding site.

It has recently been reported that pharmacological stimulation of protein kinase C (PKC) with the phorbol ester PMA activated human (and zebrafish but not mouse) TRESK current [32], [33]. The effect was reported to be independent of calcineurin [33]. In another study, application of laminar shear stress, hypotonic solution, or negative pressure on cell-attached membrane patches of trigeminal neurons activated the channel, indicating moderate mechanosensitivity [34]. These results are potentially interesting from the physiological point of view; however, it is unknown at present, which region of the channel is targeted by these activating mechanisms.

Because of the critical role played by the cytoplasmic loop of TRESK in the regulation of channel activity, we have decided to search for the interacting partners of this polypeptide by biochemical methods. In addition to the known TRESK-interacting proteins (calcineurin and 14-3-3), we detected substantial binding of tubulin to the cytoplasmic loop of TRESK.

## Materials and Methods

### Affinity chromatography

The construction of TRESK-loop-His_8_ plasmid from mouse TRESK, the expression of the protein in *E. coli* and its purification under denaturing conditions were previously described [30]. The phosphorylation of TRESK-loop-His_8_, immobilized on 1 ml Ni-NTA agarose (Qiagen, Chatsworth, CA), was performed at 37°C overnight with protein kinase A holoenzyme (PKA, Sigma P5511) in a solution containing (in mM): HEPES 50, KCl 50, MgCl_2_ 10, β-glycerol phosphate 50, imidazole 20, β-mercaptoethanol 2, sodium orthovanadate 0.2, ATP 5, cAMP 1 (pH 7.5 with NaOH), supplemented with 1% Triton X-100 and 0.02% sodium azide.

The resins, 1 ml Ni-NTA for control and 1 ml Ni-NTA with the immobilized bait were packed into columns. Chromatography was performed by using an ÄKTA FPLC system (controlled by Unicorn v3.1 software, Amersham Pharmacia Biotech). The columns were washed with 10 ml solution A containing (in mM): KH_2_PO_4_ 50, NaCl 50, imidazole 70, MgCl_2_ 2, β-mercaptoethanol 5, PMSF 1, benzamidine 1 (pH 7.0 with HCl), supplemented with 5% glycerol. Cerebrum, cerebellum and brainstem from two mice were homogenized in 5 ml solution A on ice. The lysate was centrifuged at 27,000 g for 20 min at 4°C, the supernatant was supplemented with CHAPS (to a final concentration of 1%) and centrifuged again at 27,000 g for 20 min. The cleared supernatant was loaded to the control Ni-NTA column (at 0.25 ml/min), and the flow-through from this column was loaded to the other column containing TRESK-loop-His_8_. The columns were washed with 6 ml solution A, and the proteins were eluted with a 15 ml linear gradient (0.5 ml/min) from 100% solution A to 100% solution B. (Solution B contained 2 M NaCl in addition to the components of solution A). Subsequently, the proteins remaining on the columns after the NaCl gradient were eluted with a solution containing (in mM): NaH_2_PO_4_ 30, β-mercaptoethanol 2, PMSF 1, benzamidine 1 (pH 7.0 with NaOH), supplemented with 7 M urea. Fractions of 1 ml were collected throughout the NaCl gradient and urea elution. As in the further experiments, the eluted proteins were analyzed by Tris-glycine SDS-PAGE on 10 or 12% gels, and visualized by Coomassie Brilliant Blue staining.

### Constructs for the pull-down assays

Plasmids for the different bait proteins were constructed by standard molecular biological methods. Briefly, the coding sequence of fragment 174–280 of human TRESK [11] was amplified by PCR and subcloned into our pETH8 vector containing a custom-designed multiple cloning site (MCS) and the sequence coding for the C-terminal 8 histidines, resulting in the human version of TRESK-loop-His_8_. To obtain GST fusion proteins, the insert from this vector was also subcloned into pGEX-4T1 (Amersham Biosciences, Little Chalfont, UK). This construct contained the additional AAVERPHRD amino acids at its C terminus after the TRESK coding sequence.

The coding sequence was cleaved at native restriction enzyme sites of human TRESK (BseJI, BglII and Eco130I) for the construction of fragments 174–231, 174–247, 204–280 and 232–280. Subcloning to pGEX-6P3, or self-ligation of the plasmid after cleavage with another enzyme in the MCS (followed by polishing with Klenow polymerase in some cases) were used to obtain these constructs. These clones were used as PCR-templates for the construction of the short (≈30 amino acid) fragments. Accordingly, the constructs contained some artificial sequences of amino acids between the GST and TRESK fragment (PNSL for 204–280) or at their C terminus (VERPHRD for 174–231, WSSGRIVTD for 200–231, GRAAAS for 174–247 and 218–247, ERPHRD for 204–280, AAVERPHRD for 232–280, 247–280 and 256–280). Fragments 259–280, 265–280 and 270–280 also contained C-terminal AAVERPHRD. However, fragments 256–275, 256–271 and 256–267 were free of this tag, and contained TRESK coding sequence at their C-terminus. (The binding of tubulin is independent from the C-terminal appendage, because fragment 256–280 interacts with tubulin but 270–280 does not interact, although both contain AAVERPHRD. Moreover, fragment 256–271 also binds to tubulin in the absence of the C-terminal appendage.)

The baits for testing the short (16–18 amino acid) human, *Danio* and *Gallus* TRESK motifs were constructed by inserting oligonucleotide dimers into the MCS of our pGEX-Q_10_H_8_ vector between the BamHI and XhoI sites. Thus GST was present at the N-terminal side of the peptide, whereas it was followed by the AN(Q)_10_LD(H)_8_ sequence (in single letter amino acid code) at the C-terminus.

Human TRESK-loop-His_8_ was purified in an identical manner as it was previously described for its murine counterpart [30]. GST fusion proteins were purified as previously described [30]. The GST-Q_10_H_8_ fusion proteins were eluted from the glutathione agarose and bound to Ni-NTA resin as a second round of purification.

### GST and His-tag pull-down experiments

Mouse brain cytosol was prepared for the pull-down experiments similarly as in the case of the affinity chromatography. One brain was homogenized in 2.5 ml solution A for a typical of 5–10 assays. (In GST pull-down experiments solution A without imidazole was used, whereas in His-tag pull-down assays the imidazole concentration was between 20 and 70 mM in the different experiments. In the experiments testing the competition of 14-3-3 and tubulin, solution A was supplemented with the following protease and phosphatase inhibitors: 50 mM NaF (instead of NaCl), 10 mM p-nitrophenyl-phosphate (PNPP), 4 µg/ml leupeptin, 0.2 mM sodium orthovanadate, 10 µM cyclosporin A and 0.8 µM FK506.) In order to reduce the nonspecific binding of proteins in the assays, the cytosol was preincubated with a high volume (0.5–1 ml) of the chromatographic support for 1 h at 4°C in most experiments.

The resins (5–25 µl for a reaction) with the appropriate immobilized fusion protein were washed with 1 ml solution A, and afterwards they were preincubated in most experiments with 40 µg bovine serum albumin (BSA) for 15 minutes. After this blocking of the nonspecific binding sites of the chromatographic medium, cytosol was added and the binding reaction was performed by gently rotating the beads for 1 h at 4°C. The resins were first washed with 1.3 ml of solution A. The second washing step was a high salt wash for 5 minutes with solution A containing 1 M NaCl. The final (third and fourth) washing steps were also performed with solution A to remove residual salt. The proteins were eluted from the resins with SDS sample buffer and analyzed by SDS-PAGE.

### SDTHS-PAGE

Far back in the past, α and β tubulin were distinguished on the basis of their different mobility on SDS-PAGE gels, however, several laboratories could not reproduce this separation. Finally, it has been realized [35] that the separation relies on the contaminants in some commercial SDS (sodium dodecyl sulphate) preparations, namely on sodium tetradecyl and hexadecyl sulphate (STS and SHS). Luckily, Sigma still sells its crude SDS (catalog number L5750); about 30% of L5750 is constituted by these long carbon-chain compounds according to the specification of the manufacturer. SDS was replaced by L5750 mixture for the separation of α and β tubulins in runs we called SDTHS-PAGE. In these experiments, 7.5% polyacrylamide gels were used for better resolution in the 50 kD range.

### Immunoblot analysis

SDS-PAGE of denatured samples was performed on 12% gels, and the proteins were transferred to nitrocellulose membranes (Schleicher and Schuell, Keene, NH, USA). Nonspecific binding sites of the membrane were blocked by 5% non-fat milk in PBS-T solution (phosphate buffered saline containing 1% Tween 20). The primary antibody was monoclonal anti-β-tubulin isotype III IgG (Sigma T5076) diluted 5000× in PBS-T containing 1% bovine serum albumin. The secondary anti-mouse antibody (horseradish peroxidase-conjugated IgG from goat, R05071, Advansta, Menlo Park, CA, USA) was diluted 5000× in PBS-T containing 0.5% non-fat milk. The membrane was washed once after blocking and four times after the antibodies for 5 minutes in PBS-T. The bands were visualized by the enhanced chemiluminescence detection method (WesternBright ECL HRP, Advansta) according to the manufacturer's instructions. Densitometry analysis was performed with ImageJ 1.47v software written by Wayne Rasband (Research Services Branch, NIH, Bethesda, MD, USA).

### Animals and tissue preparation

Mouse tissues derived from NMRI mouse strain (Toxicop, Hungary). *Xenopus* oocytes were prepared, the cRNA was synthesized and microinjected as previously described [11]. All treatments of the animals were conducted in accordance with state laws and institutional regulations. The experiments were approved by the Animal Care and Ethics Committee of Semmelweis University (approval ID: XIV-I-001/2154-4/2012).

## Results

### Identification of tubulin as a TRESK-interacting protein by affinity chromatography

In order to identify proteins interacting with the cytoplasmic loop of TRESK we used affinity chromatography. A part of the loop (amino acids 185–292 of mouse TRESK), extended with a C-terminal octahistidine tag, was produced in *E. coli*. Two columns were prepared. The control column (N) was packed with 1 ml Ni-NTA agarose, whereas the other column (T) contained the same support with the immobilized TRESK-loop-His_8_ protein. Brain cytosol from two mice was loaded on the control column (N) using ÄKTA FPLC system. The flow-through from column N was loaded to column T containing the bait protein. Both columns were extensively washed and processed further in the same way. Proteins were eluted with 15 ml of linear 50 mM to 2 M NaCl gradient. However, there was no apparent difference between the corresponding fractions N (from the Ni-NTA control column) and T (from the TRESK-loop column), when they were analyzed on SDS-PAGE gels (*not shown*).

**Figure 1:**
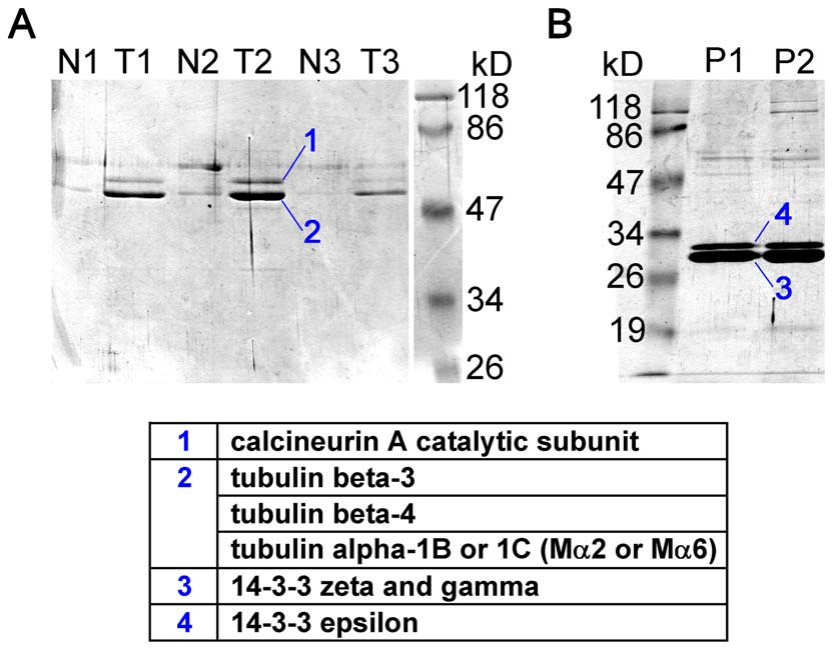
Calcineurin, tubulin and 14-3-3 are the major proteins binding to TRESK-loop-His_8_ in affinity chromatography experiments. **A.** Mouse brain proteins, remaining on the columns after the NaCl gradient, were eluted with 7 M urea. Three fractions from the Ni-NTA control column (N1–N3) and from the column containing TRESK-loop-His_8_ (T1–T3) were analyzed by SDS-PAGE and Coomassie Blue staining. The two intense bands from fraction T2 were identified by mass spectrometry as calcineurin and tubulin (as indicated in the table below the gel). **B.** TRESK-loop-His_8_ (immobilized on Ni-NTA resin) was phosphorylated with protein kinase A (PKA) before the affinity chromatography. The phosphorylated bait protein interacted with different 14-3-3 isoforms (see band 3 and 4; P1 and P2 lanes represent two independent experiments).

Subsequently, 7 M urea was applied for the elution of proteins still remaining on the columns after the NaCl gradient. Two intense bands appeared in the first three fractions eluted from column T (see T1, T2, T3 in Fig. 1.A), which were absent or much less abundant in the corresponding fractions of the control column (N1, N2, N3). Mass spectrometry analysis indicated that *band 1* was calcineurin A catalytic subunit, whereas *band 2* was identified as a mixture of different tubulin isoforms (Fig. 1.A). Tubulin β3 and β4 were unequivocally identified in the mixture, whereas the obtained peptide masses of α tubulin could correspond to both isoform α1B and α1C (also called Mα2 and Mα6 [36]). The retention of high amount of tubulin on column T but not on N indicated that tubulin associated to TRESK-loop-His_8_ protein, but much less to the chromatographic resin. The band of tubulin was substantially more intense than that of calcineurin, a known interacting protein of TRESK. The persistent attachment of tubulin and calcineurin on column T during the high salt gradient suggests that hydrophobic interactions are important in the binding of these two proteins to the intracellular loop of TRESK.

In another experiment, TRESK-loop-His_8_ was immobilized on Ni-NTA resin and phosphorylated with protein kinase A (PKA). Similar chromatography was performed with this phosphorylated TRESK loop protein as described above. The proteins binding to the PKA-phosphorylated TRESK loop are shown in Fig. 1.B as the P1 and P2 lanes representing two independent experiments. Two additional high intensity bands were observed if the bait protein was phosphorylated. Both of these bands corresponded to different 14-3-3 isoforms. Mass spectrometry analysis identified 14-3-3ζ and γ in band 3, whereas band 4 contained 14-3-3ε isoform of higher molecular weight (Fig. 1.B). Several additional low intensity bands were also apparent in *lanes P1* and *P2*. These were also analyzed by mass spectrometry (see figure S1). However, only non-specific hits (e.g. mitochondrial, nuclear, chaperone or bovine proteins) were obtained in addition to calcineurin and tubulin (in this case tubulin β2C was identified). The binding of 14-3-3 to TRESK-loop-His_8_ is in good accordance with our previous results that 14-3-3 functionally interacts with TRESK, if the channel is phosphorylated by PKA [29], [30].

### A 16 amino acid fragment of the cytoplasmic loop is sufficient for the binding of tubulin

**Figure 2:**
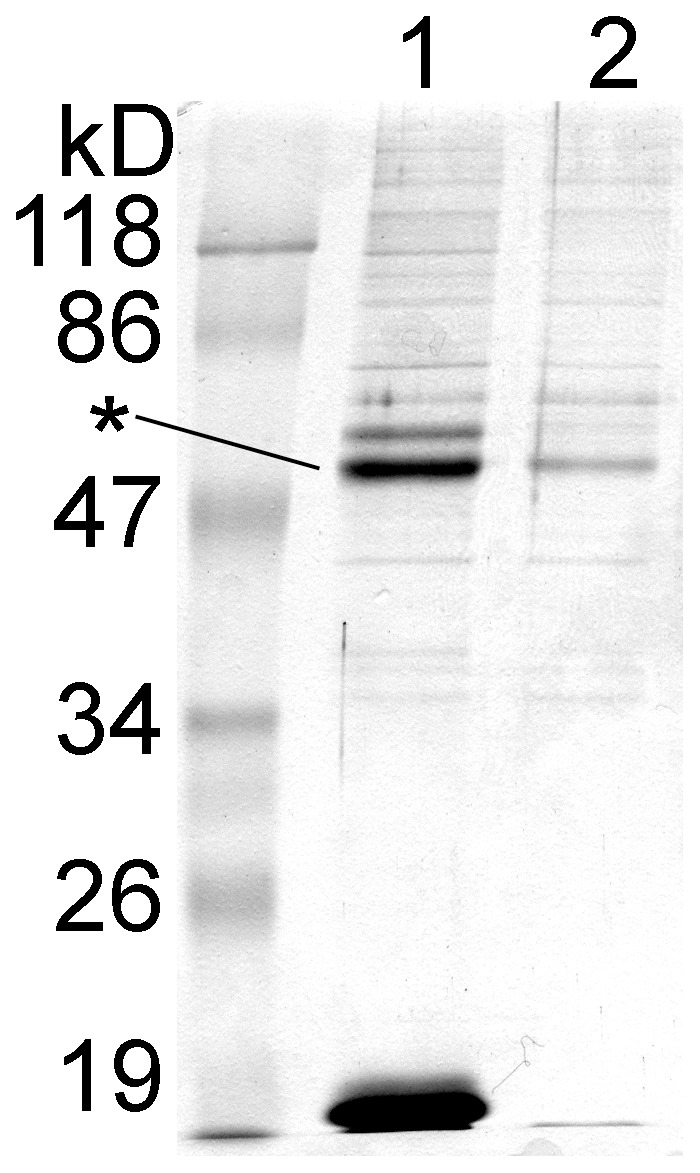
The binding of tubulin and calcineurin to the cytoplasmic loop of mouse TRESK is also reproduced in pull-down assays. Ni-NTA resin with immobilized TRESK-loop-His_8_ (*lane 1*, see the bait protein below 19 kD) or without the bait (*lane 2*) were used to pull down protein partners from mouse brain cytosol. Tubulin (indicated with an *asterisk*) clearly interacted with the bait protein, although its nonspecific binding to the control resin was also non-negligible. The band of calcineurin A subunit is discernible above that of tubulin in *lane 1*. (In this experiment, the nonspecific binding sites of the resin were not blocked with bovine serum albumin (BSA) and the cytosol was not depleted by preincubation with the chromatographic resin before the pull-down assay. These procedures were generally applied in further pull-down experiments, to reduce the nonspecific binding of proteins to the resin.)

The persistent binding of tubulin to TRESK loop during the long washing step and NaCl gradient encouraged us to also demonstrate the interaction in pull-down assays. In Fig. 2, we show the result of a pull-down assay performed with the same TRESK-loop-His_8_ protein (*lane 1*) as used in the affinity chromatography, compared to control Ni-NTA resin (*lane 2*). Although the non-specific background was high on the control resin, the binding of tubulin (indicated with an *asterisk*) and calcineurin to the bait protein was evident. One conclusion from the pull-down experiments was that tubulin has a tendency to adhere to all tested chromatographic supports. Significant efforts were made to reduce this non-specific interaction (see *Methods*); however, the adherence of tubulin to resins has not been completely eliminated. This non-specific binding limited the sensitivity of the pull-down assays for the detection of specific interactions of tubulin; therefore its degree was routinely evaluated by appropriate control (resin only, or resin only with the fusion tag) reactions.

**Figure 3:**
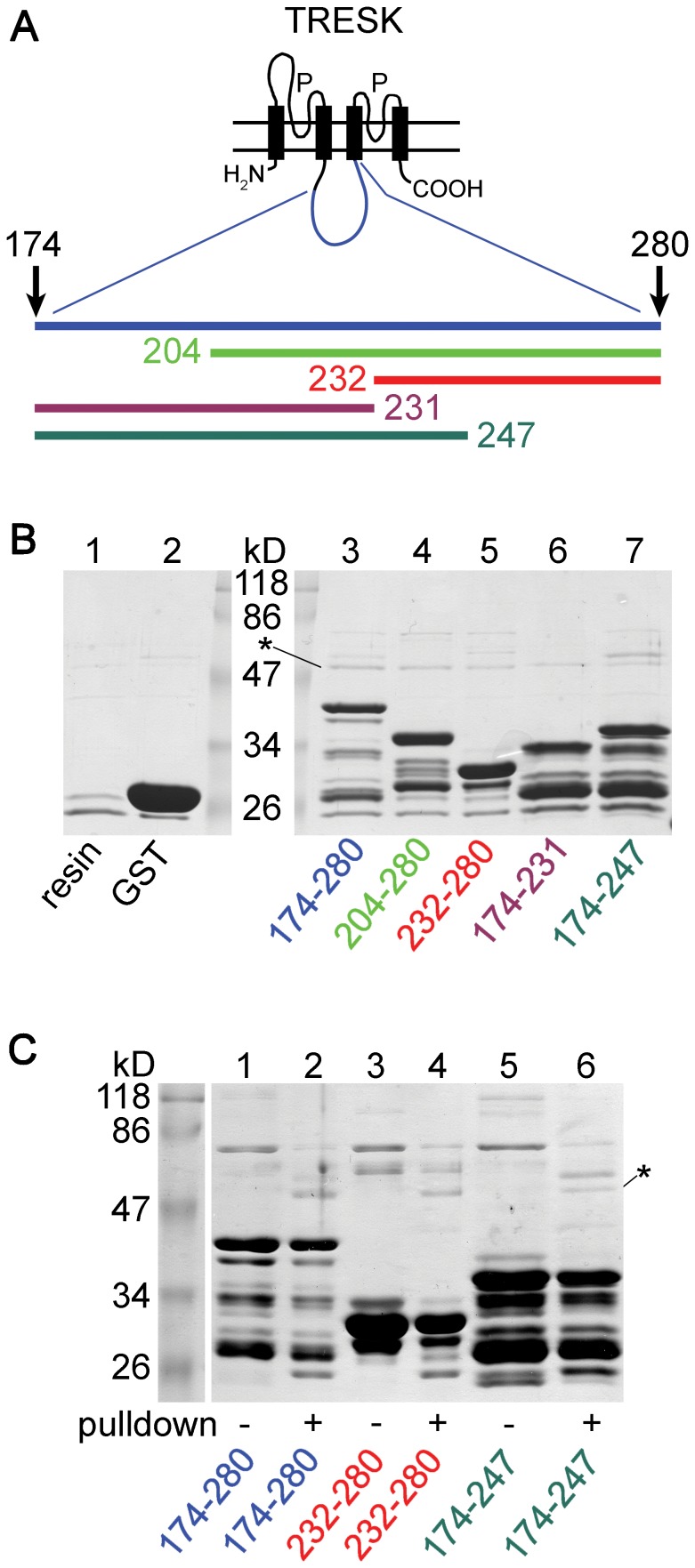
Tubulin binds to GST fusion constructs containing long fragments of the cytoplasmic loop of human TRESK. **A.** Schematic transmembrane topology of TRESK subunit is shown. The fragments of the intracellular loop (174–280, 204–280, 232–280, 174–231 and 174–247), which were fused to GST, are indicated with bars of different colors. **B.** Proteins were pulled down from mouse brain cytosol with the different GST fusion constructs (as indicated below *lanes 3–7*). All constructs interacted with tubulin (indicated with an *asterisk*). GST-TRESK-loop bait protein preparations contained several incompletely translated fragments in addition to the uppermost full-length product. (The molecular weight of full-length bait proteins was smaller than 47 kD in each cases.) Control assays were performed with glutathione agarose (*lane 1*) or with high amount of GST immobilized on the resin (*lane 2*). Pull-down of tubulin was much lower in these controls than in the assays containing TRESK fragments. (The lane of marker proteins was split to introduce size labels.) **C.** Proteins from the above pull-down assays (*even* lane numbers; as indicated with color coded labels below the gel) were compared to the corresponding bait protein preparations (*odd* lane numbers). Tubulin (indicated with an *asterisk*; *lane 2*, *4* and *6*) was pulled down from brain cytosol (similarly to calcineurin in the pull-down assays with fragments 174–280 and 174–247 in *lane 2* and *6*). In contrast to tubulin and calcineurin, which appeared only in the pull-down assays, some other bands were more intense in the bait protein preparations (e.g. the bacterial contaminant below 86 kD; see the *odd* lanes). Note that higher amount of bait was loaded in the control (*odd*) than in the pull-down (*even*) lanes, and the nonspecifically binding proteins of the bait preparations could also be removed by the washing steps in the pull-down assay.

In the following experiments we examined the interaction between TRESK loop and tubulin in a different experimental context. We used GST fusion proteins. (These could be purified from *E. coli* under native conditions in contrast to TRESK-loop-His_8_.) Accordingly, the chromatographic support was glutathione agarose instead of Ni-NTA, possibly resulting in lower tubulin-binding background. GST fusion proteins were constructed from the cytoplasmic loop of human TRESK (amino acids 174–280) to demonstrate that tubulin also interacted with the human channel. In order to search for a circumscribed tubulin-binding region within TRESK loop, shorter fragments were also examined in the pull-down assays (Fig. 3.A). (Native restriction enzyme sites of human TRESK DNA were used for cleavage during the construction of these fragments.)

Human TRESK loop (174–280) interacted with tubulin and calcineurin (similarly to its murine counterpart), in contrast to glutathione agarose and the GST-only control (Fig. 3.B, compare *lane 3* to *lanes 1* and *2*, tubulin is indicated with an *asterisk*). Since the GST fusion protein preparations also contained high molecular weight bacterial contaminants, we verified in a different SDS-PAGE run that the band corresponding to tubulin derived from mouse brain cytosol (Fig. 3.C). On this gel, a high amount of bait protein was compared to the result of the pull-down experiment (Fig. 3.C, compare *lane 1* to *2* for TRESK-loop 174–280). The tubulin and calcineurin bands are apparent only in the pull-down reaction (*lane 2*), indicating that the proteins were of cytosolic origin.

Interestingly, all the truncated fragments (amino acids 204–280, 232–280, 174–231, and 174–247; Fig. 3.B, *lanes 4–7*) interacted with tubulin. Consistent results were obtained, when the same experiment was repeated (with higher amounts of bait proteins, see figure S2). These observations indicate that the binding of tubulin to TRESK loop does not depend on a single short determinant in the sequence, but there are multiple (at least two) contact points between the two proteins. Next, we asked whether we can find at least one of these determinants of tubulin-binding in TRESK loop.

**Figure 4:**
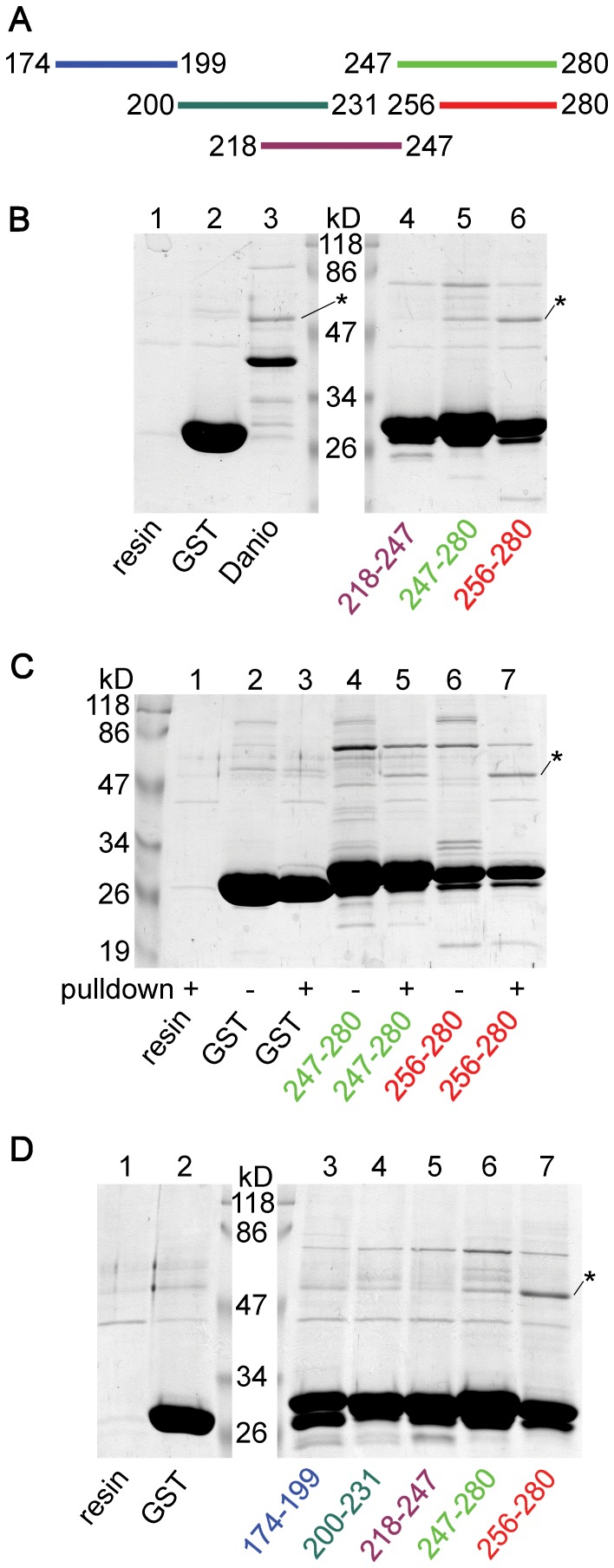
The C-terminal end of the cytoplasmic loop is a major determinant of tubulin-binding. **A.** GST fusion proteins were constructed from short (about 30 amino acid) fragments overlapping with the 174–280 region of human TRESK as indicated by the colored bars. **B.** Pull-down experiments (as in Fig. 3.B) were performed with the middle part (218–247, *lane 4*) and the C-terminal fragments (247–280 and 256–280, *lane 5* and *6*) of the cytoplasmic loop of TRESK. Tubulin interacted with the C-terminal fragments, but not with the middle part of the loop. The binding of tubulin to the C-terminal fragments was substantially stronger than that in the control reactions (compare *lane 5* and *6* to *lane 1* and *2*). In *lane 3*, tubulin was pulled down with a GST fusion construct containing the cytoplasmic loop (amino acids 174–284) of *Danio rerio* (zebrafish) TRESK. **C.** A similar control SDS-PAGE as in Fig. 3. C was performed with the C-terminal fragments (247–280 and 256–280, as indicated below the gel) to verify that the band corresponding to tubulin indeed derived from the cytosol. Note the presence of tubulin in the result of pull-down assays (*lane 5* and *7*) but not in the corresponding bait protein preparations (no pull-down, *lane 4* and *6*). The binding of tubulin to GST was much weaker (*lane 3* vs. *2*) than to the C-terminal fragments. **D.** Pull-down assays with the N-terminal fragments of the loop (174–199 and 200–231, *lane 3* and *4*) were also performed in addition to the fragments tested in panel B (*lanes 5–7*). It was reproduced that the C-terminal fragments pulled down tubulin (*lane 6* and *7*) but the middle part failed to do so (*lane 5*). Interaction of tubulin with the N-terminal fragments (*lane 3* and *4*) was weaker than that with the C-terminal fragments (*lane 6* and *7*), and was not much different from the control reactions (*lane 1* and *2*). (In panel B and D, the lane of marker proteins was split to introduce size labels. Tubulin was indicated with *asterisk*s.)

GST fusion proteins containing short (around 30 amino acid) fragments covering the 174–280 region of human TRESK were constructed (Fig. 4.A). Fragments 247–280 and 256–280, corresponding to the C-terminal part of the loop, robustly interacted with tubulin (Fig. 4.B, *lane 5* and *6*). In contrast, the middle part of the loop (218–247) did not bind tubulin (*lane 4*); or at least its binding was not stronger than that of the control resin (*lane 1*) or GST alone (*lane 2*). When the bait protein preparations were compared to the eluted proteins from the corresponding pull-down reactions, it was evident that tubulin binding to the C-terminal fragments derived from the cytosol, as illustrated in Fig. 4. C (*lane 4* vs. *5*, and *6* vs. *7*). It was reproduced in another experiment that tubulin interacted with the C-terminal fragments but not with the middle part of the loop (Fig. 4.D, *lanes 5–7*). In contrast to the above clear data, fragments 174–199 and 200–231, covering the N-terminal part of the 174–280 range, gave ambiguous results. Although their tubulin-binding appeared to be more intense than that of fragment 218–247 (Fig. 4.D, compare *lane 3* and *4* to *lane 5*), the difference between them and the controls (*lane 1* and *2*) was not convincing. Thus it is possible that these regions (174–199 and 200–231) also contain weak tubulin-binding determinants; however, this could not be proved because of the non-specific binding of tubulin to the resin in the pull-down assays. Since fragment 256–280 unequivocally interacted with tubulin, we investigated this region further.

**Figure 5:**
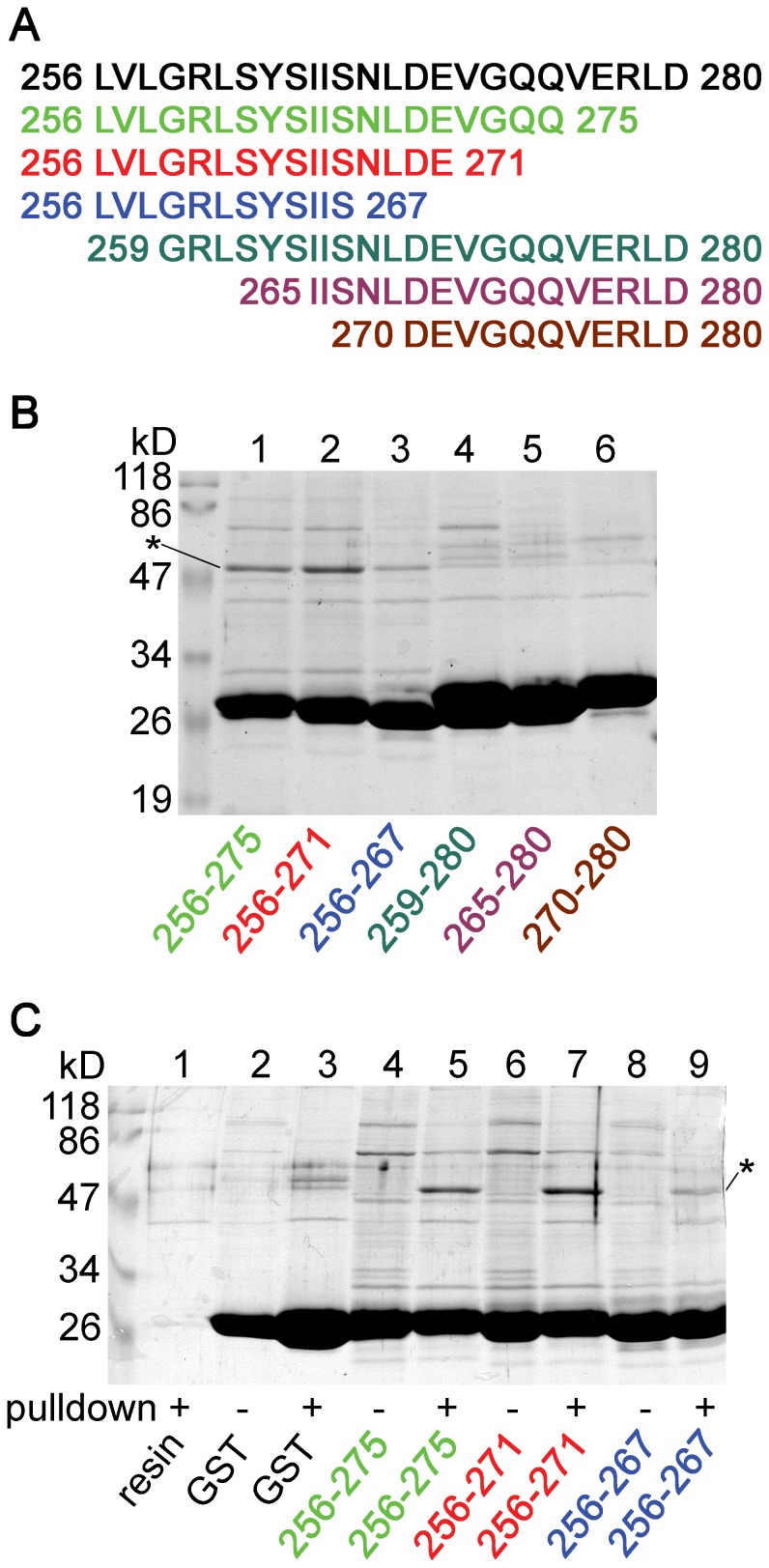
Fragment 256–271 of the cytoplasmic loop is sufficient for the binding of tubulin. **A.** The tubulin-binding fragment 256–280 of human TRESK (black, determined in the previous experiment, Fig. 4) was further truncated from the N- or the C-terminal direction as indicated by the colored sequences. **B.** Pull-down experiment was performed (as in Fig. 3 and 4) with the GST fusion constructs containing the sequences indicated in panel A and also below the lanes. Fragment 256–275 (*lane 1*) and 256–271 (*lane 2*) robustly interacted with tubulin. Tubulin was pulled down less avidly by fragment 256–267 (*lane 3*) than by the latter two fragments, whereas the N-terminal truncations (fragments 259–280, 265–280 and 270–280, *lane 4–6*) diminished the binding of tubulin. **C.** Proteins from the pull-down assays were compared to the bait preparations (as in Fig. 3.C and 4.C). Note the substantial amount of tubulin binding to fragments 256–275 and 256–271 (*lane 5* and *7*), in sharp contrast to the *lanes* (*4* and *6*) of the baits. (Tubulin was indicated with an *asterisk*.)

Series of truncations were performed from both the N- and C-terminal directions to approximate the minimally required sequence for the binding of tubulin (Fig. 5.A). Fragments 256–275 and 256–271 substantially interacted with tubulin (Fig. 5.B, *lane 1* and *2*). Tubulin bands proved to be of cytosolic origin also in these reactions (Fig. 5.C, *lane 4* vs. *5* and *6* vs. *7*). Fragment 256–267 still attracted tubulin, although less avidly than fragment 256–271 (Fig. 5.B, compare *lane 2* to *3*). In contrast, N-terminal truncations (fragments 259–280, 265–280 and 270–280) diminished the binding of tubulin (Fig. 5.B, *lanes 4–6*). Therefore we concluded that fragment 256–271 is a major determinant of the interaction of tubulin with the cytoplasmic loop of TRESK.

**Figure 6:**
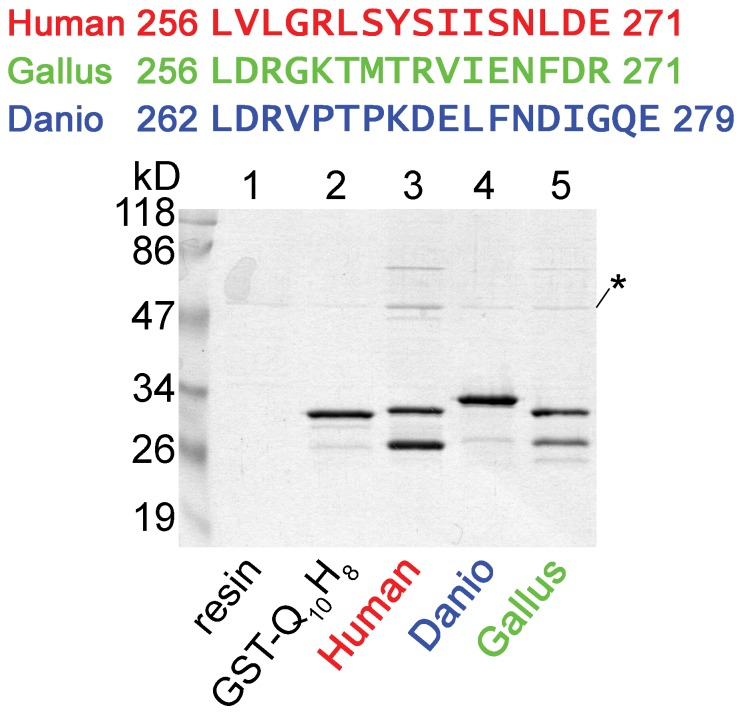
The fragments of zebrafish and chicken TRESK, homologous to the tubulin-binding motif of the human channel, do not efficiently interact with tubulin. Fragments of TRESK from *Danio rerio* (zebrafish, 18 amino acids) and *Gallus gallus* (chicken, 16 amino acids), homologous to region 256–271 of the human channel (as shown on the top of the figure), was tested in pull-down assays. Neither the fragment from *Danio* (*lane 4*) nor that from *Gallus* (*lane 5*) interacted with tubulin so strongly as the tubulin-binding motif of human TRESK (*lane 3*). In this experiment, the short peptide fragments were inserted between N-terminal GST and C-terminal Gln_10_His_8_ tags, thus they were not located at the extreme C-terminus of the fusion proteins. Ni-NTA resin (*lane 1*) and GST-Gln_10_His_8_ with no insert (GST-Q_10_H_8_, *lane 2*) were used as controls.

The cytoplasmic loop of the distantly related *Danio rerio* (zebrafish) TRESK (amino acids 174–284) also binds tubulin (as a GST-fusion protein, Fig. 4.B, compare *lane 3* to *lanes 1* and *2*). However, the tubulin-binding sequence, identified in the human channel, is not highly conserved in *Danio* TRESK (Fig. 6). We examined whether this region of the fish channel, analogous to the 16 amino acid human sequence, binds to tubulin or not. (The fragment of *Danio* TRESK was extended by two amino acids, because of the low homology between the human and fish channels at the C-terminal boundary of the region.) We also tested a more closely related homolog of TRESK from *Gallus gallus* (chicken). Neither the chicken nor the fish sequence interacted with tubulin to the extent characteristic for the fragment of the human channel (Fig. 6). Thus *Danio* TRESK contains (an)other sequence determinant(s) for the interaction with tubulin, and the 16 amino acid tubulin-binding region is characteristic for the mammalian TRESK channel.

### The cytoplasmic loop of TRESK binds approximately equal amounts of α and β tubulin

Tubulin was identified by mass spectrometry from the proteins obtained with affinity chromatography. Apparently the same bands were detected in the pull-down experiments, although tubulin has not been explicitly identified in these reactions apart from its molecular weight. Thus, formally, additional evidence is required that the protein around 50 kD was indeed tubulin in the pull-down assays. In addition, we were curious whether there is a preference of TRESK loop for the binding of α or β tubulin. In order to address these two issues simultaneously, we decided to separate α and β tubulin on SDTHS-PAGE gels (see *Methods*).

**Figure 7:**
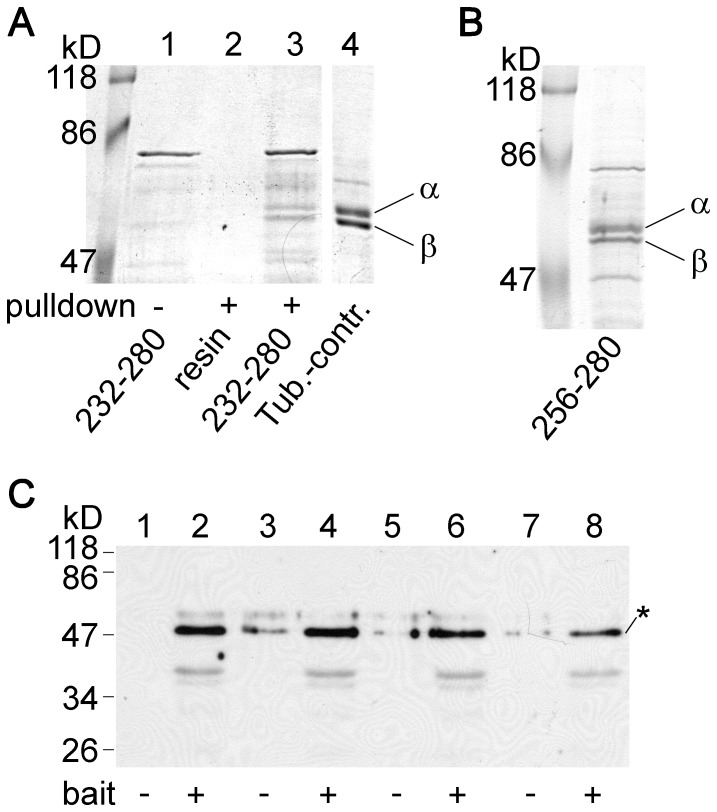
Tubulin interacting with the cytoplasmic loop of TRESK migrates as a double band on SDTHS-PAGE gel. **A.** The proteins from the pull-down assay with fragment 232–280 of human TRESK were analyzed by SDS-PAGE in the presence of STS and SHS. Under these special conditions, the band split into a doublet of α and β tubulins (*lane 3*), although the same protein migrated as a single band on normal SDS-PAGE gels (see in Fig. 3.B *lane 5* or Fig. 3.C. *lane 4*). The double band could be detected neither in the bait protein preparation (*lane 1*) nor in the control pull-down assay with glutathione agarose (*lane 2*). **B.** Similar splitting of the band was observed on SDTHS-PAGE gels in the case of tubulin pulled down with fragment 256–280 of human TRESK. **C.** Western blot experiment with monoclonal anti-tubulin β3 antibody was performed from four pairs of independent pull-down assays. The analyzed proteins were pulled down from mouse brain cytosol with the GST fusion protein containing residues 174–280 of human TRESK (*even* lanes, *bait +*) or with glutathione agarose (*odd* lanes, *bait −*). The anti-tubulin β3 antibody specifically labeled the tubulin bands (indicated with an *asterisk*). Densitometry and statistical analysis were performed as detailed in Fig. S3.

The band from the pull-down assay of fragment 232–280, running as a single entity around 50 kD on normal SDS-PAGE gels, split into a doublet under the specific conditions of SDTHS-PAGE (Fig. 7.A, *lane 3*). Similar splitting was observed in the case of the protein pulled down with fragment 256–280 (Fig. 7.B, *lane 1*). In sharp contrast, the other proteins (e.g. the bacterial contaminant below 86 kD, or the marker proteins) migrated as single bands also on SDTHS-PAGE gels. Splitting of the band under SDTHS-PAGE conditions is a strong argument in favor of the hypothesis that the band represents tubulin. Together with the mass spectrometry data of the corresponding band from affinity chromatography, we consider it as proof that the cytoplasmic loop of TRESK interacted with tubulin in the pull-down assays.

The intensity of the two daughter bands, those of the low mobility α and high mobility β tubulins [35], appeared to be equal (Fig. 7. A and B). The most plausible explanation of this result is that the functional unit of tubulin, the α–β heterodimer associates to TRESK. Nevertheless, the binding of separate subunits also cannot be ruled out, if TRESK loop discriminates poorly between α and β tubulin.

In order to further verify and also statistically evaluate the binding of tubulin to the cytoplasmic loop of TRESK, we performed four pairs of independent pull-down assays from mouse brain cytosol with either the bait protein (fragment 174–280) or only the control resin. The proteins were analyzed on standard SDS-PAGE gels stained with Coomassie Blue (figure S3) or with anti-tubulin β3 Western blot (Fig. 7. C). Densitometry analysis of the gel stained with Commassie Blue indicated that significantly higher amount of tubulin interacted with the bait protein than with the resin (Fig. S3, p<10^−5^, Student's t-test). The monoclonal anti-tubulin β3 antibody specifically labeled the tubulin bands and densitometry of the immunoblot also verified that the binding of tubulin to the bait protein exceeded the nonspecific background on the resin (Fig. 7.C, 20470±2835 vs. 4615±1178 counts for the bait and control reactions, respectively, n = 4 in both groups, p<0.005, Student's t-test).

### The adaptor protein 14-3-3 competes with tubulin for the binding to TRESK

Proximity of the 14-3-3 and tubulin binding sites (RSNSCPE*LVLGRLSYSIISNLDE*) prompted us to investigate the possible interference between the docking of these two proteins to the cytoplamic loop of TRESK. The association of 14-3-3 to TRESK depends on the phosphorylation of a serine (*underlined*, see the above sequence) in the channel [30]. Therefore, we initially tested the binding of tubulin to TRESK loop, if this serine was or was not phosphorylated with protein kinase A before the usual pull-down assay. However, only weak binding of 14-3-3 to the PKA-treated bait was detected under these conditions (*not shown*). The bait might have been dephosphorylated by brain cytosol (e.g. by calcineurin localized to TRESK loop) before the association of 14-3-3, despite the phosphatase inhibitors included in the reaction. To ameliorate this problem, we preloaded the PKA-phosphorylated bait with 14-3-3 in advance of the pull-down assay. Bacterial supernatant containing recombinant 14-3-3 without a fusion tag was used for preloading the bait, to avoid possible steric interference of the fusion tag with the binding of tubulin. The bacterial supernatant has not been removed from the resin before the addition of mouse brain cytosol to ensure high [14-3-3] throughout the binding phase of the pull-down experiment.

**Figure 8:**
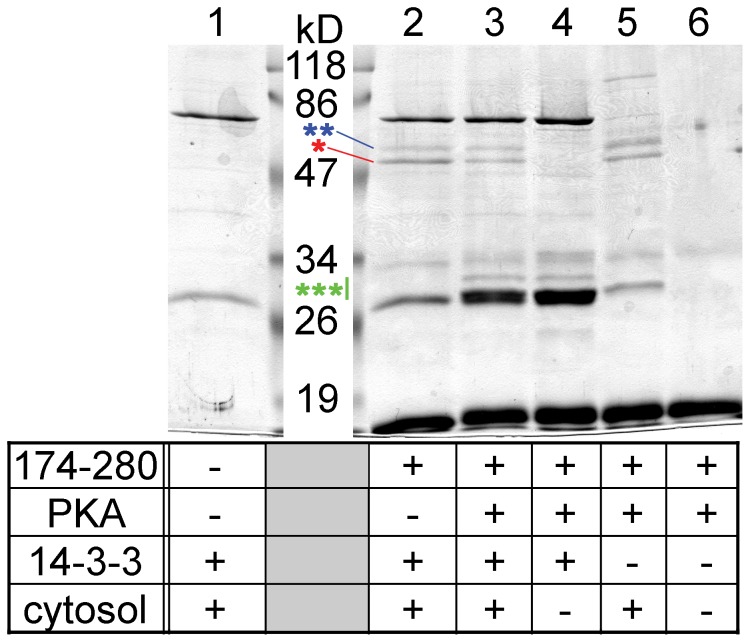
The adaptor protein 14-3-3 competes with tubulin for the binding to TRESK. TRESK-loop-His_8_ (amino acids 174–280 of the human channel; immobilized on Ni-NTA resin) was (*lane 3*) or was not (*lane 2*) phosphorylated with protein kinase A (PKA). The bait was incubated with supernatant from E. coli expressing 14-3-3η adaptor protein without fusion tag. Subsequently, mouse brain cytosol was added, and pull-down assay was performed. The binding of 14-3-3 to the phosphorylated bait is apparent in the 30 kD range (as indicated with a *green triple asterisk*; compare *lane 3* to *2*). Calcineurin (*blue double asterisk*) interacted identically with the non-phosphorylated (*lane 2*) and the 14-3-3-preloaded bait (*lane 3*). In contrast, more tubulin (indicated with a *red asterisk*) was pulled down by TRESK-loop-His_8_ with no bound 14-3-3 (*lane 2*) than by the bait preloaded with the adaptor protein (*lane 3*). Several control reactions were also performed (as indicated in the table below the gel) to demonstrate that the bands corresponding to tubulin and calcineurin were of cytosolic origin (*lanes 2* and *3* vs. *4*), to determine the source of 14-3-3 binding to TRESK-loop-His_8_ (bacterial supernatant or brain cytosol, *lane 3* vs. *5*), to identify the bands belonging to the bait and PKA preparations (*lane 6*) and to illustrate the non-specific interactions of Ni-NTA resin in this experiment (*lane 1*).

Four variables were changed (+ or −) in the reactions: the presence or absence of the bait (human TRESK-loop-His_8_ protein including amino acids 174–280 or only Ni-NTA resin), phosphorylation with PKA, preload with 14-3-3, and pull-down from mouse brain cytosol (corresponding to the four rows in the table of Fig. 8). The only difference between the reactions in *lane 2* and *3* was designed to be the phosphorylation with PKA (Fig. 8). Phosphorylation increased the binding of 14-3-3 to human TRESK-loop-His_8_ (*lane 3*, see the region indicated by *green triple asterisk*), compared to the non-phosphorylated bait protein (*lane 2*). A minor fraction of 14-3-3 originated from mouse brain cytosol (compare *lane 3* to *5*), but the majority was recombinant 14-3-3 from the bacterial supernatant. The binding of 14-3-3 in response to the phosphorylation with PKA was evident despite of the non-specific interaction of a protein with the Ni-NTA resin in the same molecular weight range (*lane 1*).

The pull-down of tubulin was reduced by the binding of 14-3-3 to TRESK loop (Fig. 8, *lane 3*, *red asterisk*), compared to the bait without the adaptor protein (*lane 2*). In sharp contrast, calcineurin equally interacted in the two reactions (*blue double asterisk*, *lane 2* vs. *3*). The bands of tubulin and calcineurin were clearly of cytosolic origin (*lanes 2* and *3* vs. *4*). Similar results were obtained when the experiment was repeated (figure S4). These data suggest that 14-3-3 competes with tubulin for the binding to the cytoplasmic loop of TRESK.

## Discussion

The general molecular architecture of two-pore domain (K2P) K^+^ channels is characterized by four transmembrane segments and two pore-loop forming domains (*P*, Fig. 3.A) in each subunit of the functional dimer. Most members of the K2P family contain a short (≈25–30 amino acid) cytoplasmic loop between the second and third transmembrane regions. In these channels (e.g. of the TASK and TREK subfamilies), the long (≈100–150 amino acid) intracellular C-terminal tail is involved in a wide variety of regulatory mechanisms, including mechanosensitivity [37], [38], modulation by intacellular pH [39], [40], phosphorylation by protein kinases [41], [42], and non-enzymatic protein-protein interactions [43]–[47].

TRESK channel possesses long (>100 amino acid) cytoplasmic loop but relatively short (≈25–30 amino acid) C-terminus. Regarding the pivotal role of the proximal region (≈30 amino acids) of the C-terminal tail in the regulation of TASK and TREK channels [38], [48], and assuming a general scheme of gating modulation by this region in the K2P family [49], it seems probable that TRESK can also be regulated via its C-terminus. However, the amino acid sequence of the C-terminal tail of TRESK significantly differs from that of the other K2P channels. Factors affecting TRESK via its C-terminus have not yet been reported.

The unique calcium-dependent activation of TRESK relies on the long cytoplasmic loop of the channel, a distinguishing structural element in the K2P family [11]. The NFAT-like calcineurin-binding site (PQIIIS) is located nearly in the middle of this loop [28], [50]. The three regulatory serines, thought to be responsible for the inhibition of TRESK [29], are concentrated at the C-terminal end of the loop, adjacent to the third transmembrane segment, within the RSNSCPELVLGRLSYSIISNLDE sequence. The first (*underlined*) regulatory serine in this sequence is phosphorylated by PKA and binds 14-3-3 both *in vitro* and in *Xenopus* oocytes [29], [30]. We have recently reported that microtubule-affinity regulating kinases (MARK kinases) phosphorylate the second and/or third (*underlined*) inhibitory serines in mouse TRESK sequence, RSNSCPELVLGRLSCSILSNLDE, *in vitro*. The coexpression of MARK kinases with human or mouse TRESK in *Xenopus* oocytes inhibited the basal activity of the channel and also accelerated the return of the K^+^ current to the resting state after the calcium-dependent activation [31]. Thus the effect of MARK on TRESK was demonstrated both *in vitro* and in the living cell. Several other kinase types (more than 20 were tested) failed to affect TRESK current via the regulatory serines phosphorylated by MARK [31]. The specificity of MARK in the regulation of TRESK raised the suspicion that the channel may be related to the microtubule cytoskeleton, an established target of microtubule-affinity regulating kinases [51].

Tubulin, the building block of microtubules, interacted with the cytoplasmic loop of TRESK in the affinity chromatography experiment of the present study. Tubulin β3 and β4 (β4A) was identified by mass spectrometry. These two beta tubulin isoforms are highly and specifically expressed in the nervous system; tubulin β3 is a frequently used neuron-specific marker. Tubulin β2C (also called β4B, a ubiquitously expressed isoform) was also detected when the bait protein was phosphorylated with PKA before the affinity chromatography (see *band 9* in *lane P2* of panel B in the figure S1). However, this result should be interpreted with caution because *band 9* was of low intensity, and a significant fraction of it may have derived from the nonspecific binding of tubulin to Ni-NTA resin. The extremely anionic C-terminal peptides of tubulins have not been detected by mass spectrometry, and thus the obtained peptide masses of α-tubulin could correspond to both α1B and α1C isoforms. Because α1B (Mα2) is a dominant isoform in the brain but α1C (Mα6) is expressed in trace amounts in this tissue [36], α1B is likely to contribute to the tubulin-binding of TRESK loop.

Approximately equal amounts of α and β tubulins were detected when the proteins from the pull-down experiments were separated on SDTHS-PAGE gels. This is in good accordance with the idea that the functional unit of tubulin, the α–β heterodimer participates in the interaction. The dissociation of the tubulin dimer is very slow *in vitro* [52]. Accordingly, the interaction of TRESK loop with only one of the subunits may be sufficient to result in the observed 1∶1 ratio of α and β tubulins. It remains to be established whether only one or both of the subunits interact directly with the cytoplasmic loop of TRESK.

The intracellular loop contains more than one short polypeptide segments responsible for the binding of tubulin. Sensitivity of the pull-down assay allowed the identification of the most prominent one as the LVLGRLSYSIISNLDE sequence in human TRESK. The sequence is reasonably well conserved between mammals, but not in lower-order organisms such as birds or fishes. Accordingly, the same region of the intracellular loop of chicken and zebrafish TRESK failed to appreciably bind tubulin in our pull-down assays. Further studies are required to determine whether a tubulin-binding consensus motif is hidden in the 16 amino acid sequence of human TRESK and other instances of this motif also mediate the interaction of different proteins with tubulin or the binding site is only specific for the channel.

We provided evidence suggesting that the PKA-dependent docking of 14-3-3 interfered with the binding of tubulin to TRESK. Some remaining ambiguities of this result follow from the complexity of the experimental approach used for the detection of the competition. TRESK loop was not saturated with 14-3-3, probably because of its dephosphorylation by the cytosol (compare the intensity of 14-3-3 bands around 30 kD to that of the bait protein below 19 kD in *lane 3* of Fig. 8). The incomplete saturation of the bait with 14-3-3 explains that the binding of tubulin was only slightly reduced but not abolished. In fact, the reduced interaction of TRESK loop with tubulin could have been caused by either the binding of 14-3-3 to the loop or the phosphorylation of the 14-3-3-binding site by PKA in this experiment. Nevertheless, we believe that the association of the bulky 14-3-3 is a more probable candidate for the effect than the phosphorylation.

We have also attempted to verify the interaction between TRESK and microtubules in the living cell. We tested paclitaxel (taxol, a microtubule-stabilizing drug) and the depolymerizing agents, colchicine and nocodazole on TRESK expressed in *Xenopus* oocytes [53], [54]. However, the K^+^ current and the calcium-dependent regulation after the pretreatment with these compounds were indistinguishable from the controls (*not shown*). We also tried to detect the physical association of microtubules to TRESK loop. A green fluorescent protein (GFP)-TRESK-loop fusion construct was expressed in HEK293 cells, but it did not highlight microtubule bundles (either under control conditions or in the presence of taxol, *not shown*). This is in contrast to the reported localization of GFP fusion constructs composed of other microtubule-binding proteins [55], [56].

We have cloned tubulin β3, β4, α1B and α1C, and coinjected high amounts of their cRNAs in different combinations after that of TRESK into *Xenopus* oocytes. We also tested tubulin β3 modified at the N-terminus from MREIV to MSSIV in order to prevent the degradation of cRNA by an autoregulatory mechanism operating at the translational level [57]. In other experiments, TRESK was coexpressed with MSSIV-tubulin β3 and tubulin α1B, both truncated at their C-terminus. The expressed amount of these α and β subunits of slightly lower molecular weight proved to be comparable to the level of endogenous tubulins in *Xenopus* oocytes. However, TRESK has not been influenced by the coexpression of the different tubulin constructs (*not shown*).

The above pharmacological and overexpression experiments did not shed light on the functional relevance of the interaction between TRESK and tubulin. Other approaches and different methodology may be required in the future for the detection and elucidation of the interaction in the living cell. Despite of the unchanged current and regulation of the channel in the presence of the microtubule stabilizing or disrupting agents in the oocytes, it is still possible that microtubules bind to TRESK but the channel activity is not influenced by this interaction. Microtubules are known to be important determinants of channel trafficking and plasma membrane localization. Well-established examples are the glycine- and GABA_A_-receptor ligand-gated ion channels, which are linked to microtubules by the gephyrin and GABARAP tubulin-binding proteins at the postsynaptic density [58]–[60]. Although in these cases aggregates of scaffolding proteins interconnect the channels to the microtubule cytoskeleton, it has been suggested that some other channels may directly interact with microtubules.

TRPV1 (Transient Receptor Potential Vanilloid subtype 1) and P2X2 purinergic receptor are two channel types expressed abundantly in dorsal root ganglion neurons similarly to TRESK, and reported to be connected to the microtubule network [61]–[63]. TRPV1 contains two polybasic regions in its intracellular C-terminus, and these may interact electrostatically with the negatively charged tubulin C-termini [61]. Thus the tubulin-binding mechanism of TRPV1 is clearly different from that of TRESK. The tubulin-binding region of P2X2 receptor was confined to a 42 amino acid long region [64]. Interestingly, the middle of this region includes the LVLGQI sequence, which is similar to the first six LVLGRL amino acids of the tubulin-binding determinant of TRESK identified in the present study. Considering that the probability of finding four consecutive identical residues in two random sequences of 16 and 42 amino acids is less than 0.005, it seems unlikely that the occurrence of LVLG in both the P2X2 receptor and TRESK tubulin-binding sites is a coincidence. P2X2 channel and TRESK may bind tubulin with a similar mechanism.

After a decade of investigation, it is still not certain whether TRPV1 and P2X2 receptor bind to microtubules or only to soluble tubulin dimers [62]–[65]. The architectural complexity level of tubulin (e.g. monomer, dimer, oligomer, protofilament, microtubule) interacting with TRESK also has not yet been defined. If TRESK binds only to α–β heterodimers but not to microtubules, then the functional role of the interaction may be different from microtubule-dependent localization and traffic. It is tempting to speculate that tubulin localized to the channel complex may be related to the inhibitory effect of microtubule-affinity regulating kinase (MARK) on TRESK [31]. Inhibition of TRESK by the three heterologously expressed MARK kinases (MARK1–3) is unequivocal [31]. Although their major substrates are microtubule-associated proteins (e.g Tau or MAP2), it is not clearly established in the literature, how these three MARK kinases are localized to tubulin [51].

Our results also hint at the possibility that the interaction between TRESK and tubulin is conditional. Phosphorylation of TRESK and the consequent anchoring of 14-3-3 may occlude the tubulin-binding site of TRESK, and prevent the association of tubulin to the channel under resting conditions. We have previously shown that 14-3-3 substantially modulates the calcium-dependent regulation of TRESK [29], suggesting that a major fraction of the channels is bound to the adaptor protein in *Xenopus* oocytes. If 14-3-3 really competes with tubulin for the binding to TRESK in the living cell, then tubulin can associate only to the activated channel when 14-3-3 is absent from its docking site because of dephosphorylation.

In summary, we provide the first evidence that the cytoplasmic loop of TRESK interacts with tubulin *in vitro*. The functional relevance of the interaction, as a challenging problem, remains to be examined.

## Supporting Information

Figure S1 **Proteins were identified by mass spectrometry analysis from the low intensity bands of TRESK-loop-His_8_ affinity chromatography.** Several bands (1–11, also including the low intensity ones) were analyzed by mass spectrometry from the same gels as shown in Fig. 1. Apart from the interacting proteins discussed in the Results section, apparently non-specific hits were obtained. Acyl-CoA-dehydrogenase, HSPD1 and ATP synthetase β subunit are mitochondrial proteins. HNRNP U is nuclear. Because of their localization, their interaction with the channel is unlikely. Bovine glycogen phosphorylase was identified by mass spectrometry. Probably it derived from the (commercial) bovine PKA enzyme preparation used for the phosphorylation of the bait. Although the specific interaction of TRESK with α-actin and DEAH box polypeptide 9 cannot be unequivocally excluded, we think that the robust expression of actin in the cell may explain its pull-down in trace amounts, and the specific interaction of TRESK with the transcriptional regulator RNA helicase DEAH box polypeptide 9 also seems to be unlikely.
(TIF)

Figure S2 **The experiment in** **Fig. 3. B** **was repeated with high amounts of bait proteins.** Pull-down of tubulin by long fragments of the cytoplasmic loop of human TRESK (as indicated below the gel) was reproduced in this experiment with high amount of bait proteins. Tubulin clearly interacted with the fragments of TRESK (*lanes 3–7*, indicated with an *asterisk*), but much less with the chromatographic resin (*lane 1*) or GST (*lane 2*). (Note that the GST control contains an intense nonspecific band of slightly higher molecular weight than that of tubulin.)
(TIF)

Figure S3 **Statistical evaluation of the binding of tubulin to the cytoplasmic loop of TRESK by Coomassie Blue staining and densitometry.** **A.** Four pairs of independent pull-down assays were performed from mouse brain cytosol with the GST fusion protein containing residues 174–280 of human TRESK (*even* lanes) or with glutathione agarose (*odd* lanes). The proteins were separated by SDS-PAGE and stained with Coomassie Brilliant Blue. Tubulin is indicated with an *asterisk*. **B.** Tubulin bands were analyzed by densitometry. The densitometry curve from ImageJ software is illustrated under the bands. **C.** The counts calculated from the densitometry curve without background subtraction are shown as a column diagram with error bars (S.E.) for the control resin and fragment 174–280 reactions. Significantly higher amount of tubulin interacted with the bait protein than with the control glutathione agarose (p<10^−5^, Student's t-test). In the bars, sample numbers are shown.
(TIF)

Figure S4 **The experiment in** **Fig. 8** **was reproduced.** The experiment shown in Fig. 8 was repeated, and practically the same result was obtained. Note that the band in the 30 kD range (indicated with a *green triple asterisk*) in *lane 2* was more intense than the nonspecific band in the Ni-NTA control reaction (*lane 1*) in this experiment. This suggests that a low amount of 14-3-3 bound to the bait even if it had not been phosphorylated with PKA in advance. The presence of protein kinases and ATP in the brain cytosol may explain the limited phosphorylation of TRESK-loop-His_8_ and the binding of 14-3-3 in this case. The bait proteins (and the 19 kD marker band) were allowed to run out of the gel during electrophoresis in this experiment to obtain higher resolution in the 50 kD range.
(TIF)
