# Supplementary figures and images for: Tubulin Binds to the Cytoplasmic Loop of TRESK Background K+ Channel In Vitro
Source: PLoS One. 2014 May 15;9(5):e97854. doi: 10.1371/journal.pone.0097854 (PMC4022642; doi:10.1371/journal.pone.0097854)

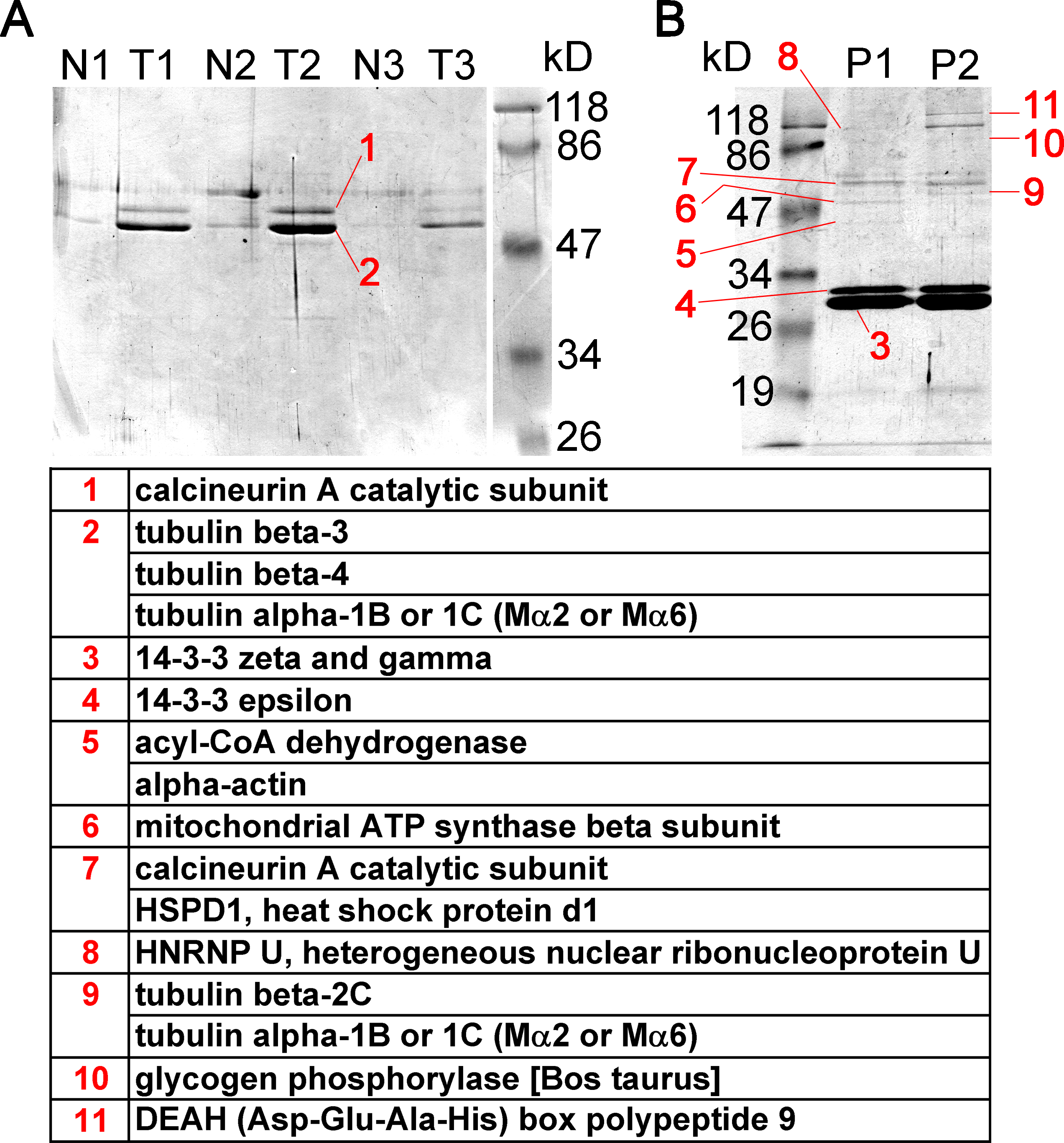

Supplement: Figure S1 — Proteins were identified by mass spectrometry analysis from the low intensity bands of TRESK-loop-His8 affinity chromatography. Several bands (1–11, also including the low intensity ones) were analyzed by mass spectrometry from the same gels as shown in Fig. 1. Apart from the interacting proteins discussed in the Results section, apparently non-specific hits were obtained. Acyl-CoA-dehydrogenase, HSPD1 and ATP synthetase β subunit are mitochondrial proteins. HNRNP U is nuclear. Because of their localization, their interaction with the channel is unlikely. Bovine glycogen phosphorylase was identified by mass spectrometry. Probably it derived from the (commercial) bovine PKA enzyme preparation used for the phosphorylation of the bait. Although the specific interaction of TRESK with α-actin and DEAH box polypeptide 9 cannot be unequivocally excluded, we think that the robust expression of actin in the cell may explain its pull-down in trace amounts, and the specific interaction of TRESK with the transcriptional regulator RNA helicase DEAH box polypeptide 9 also seems to be unlikely. (TIF) [file pone.0097854.s001.tif]

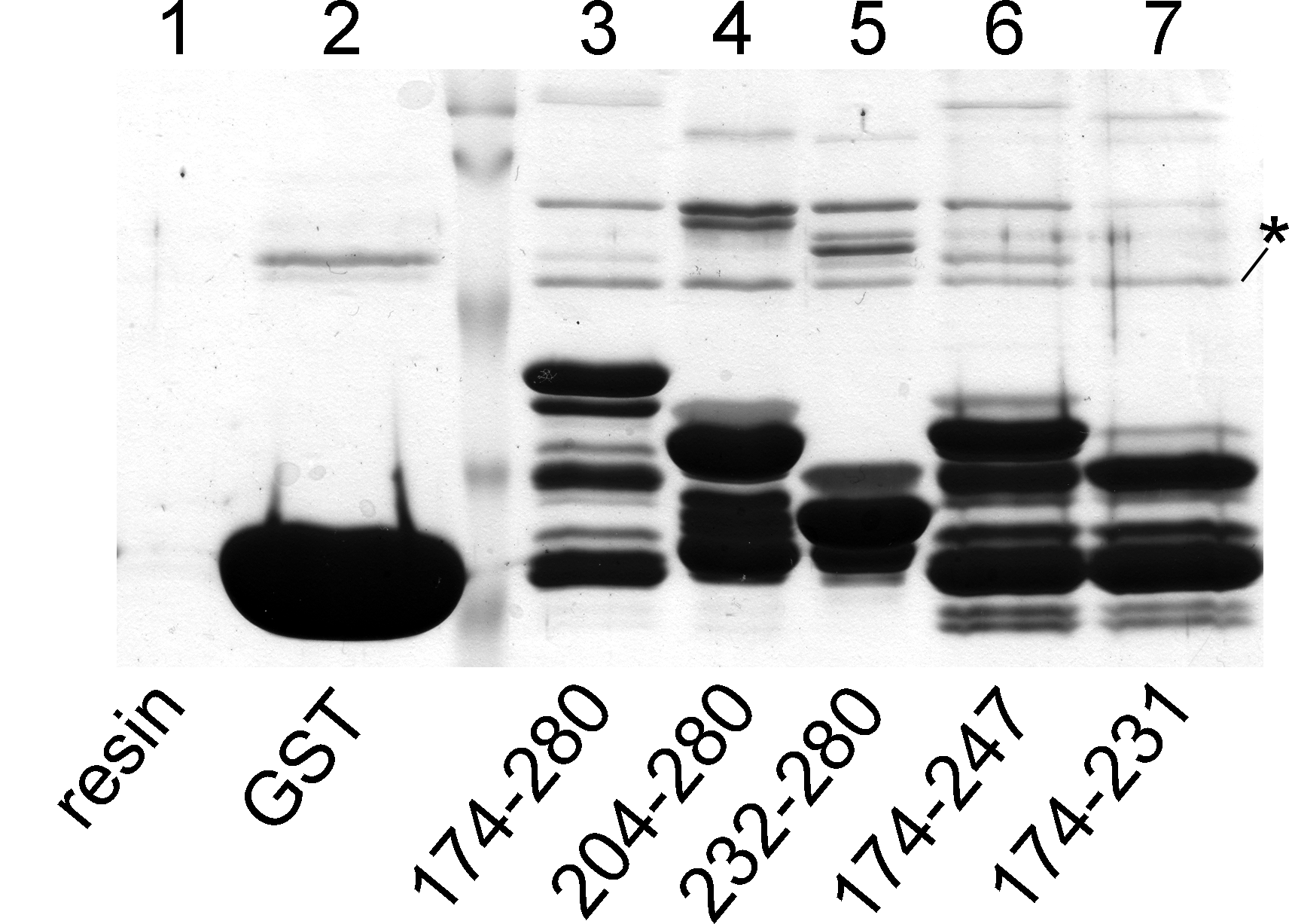

Supplement: Figure S2 — The experiment in Fig. 3. B was repeated with high amounts of bait proteins. Pull-down of tubulin by long fragments of the cytoplasmic loop of human TRESK (as indicated below the gel) was reproduced in this experiment with high amount of bait proteins. Tubulin clearly interacted with the fragments of TRESK (lanes 3–7, indicated with an asterisk), but much less with the chromatographic resin (lane 1) or GST (lane 2). (Note that the GST control contains an intense nonspecific band of slightly higher molecular weight than that of tubulin.) (TIF) [file pone.0097854.s002.tif]

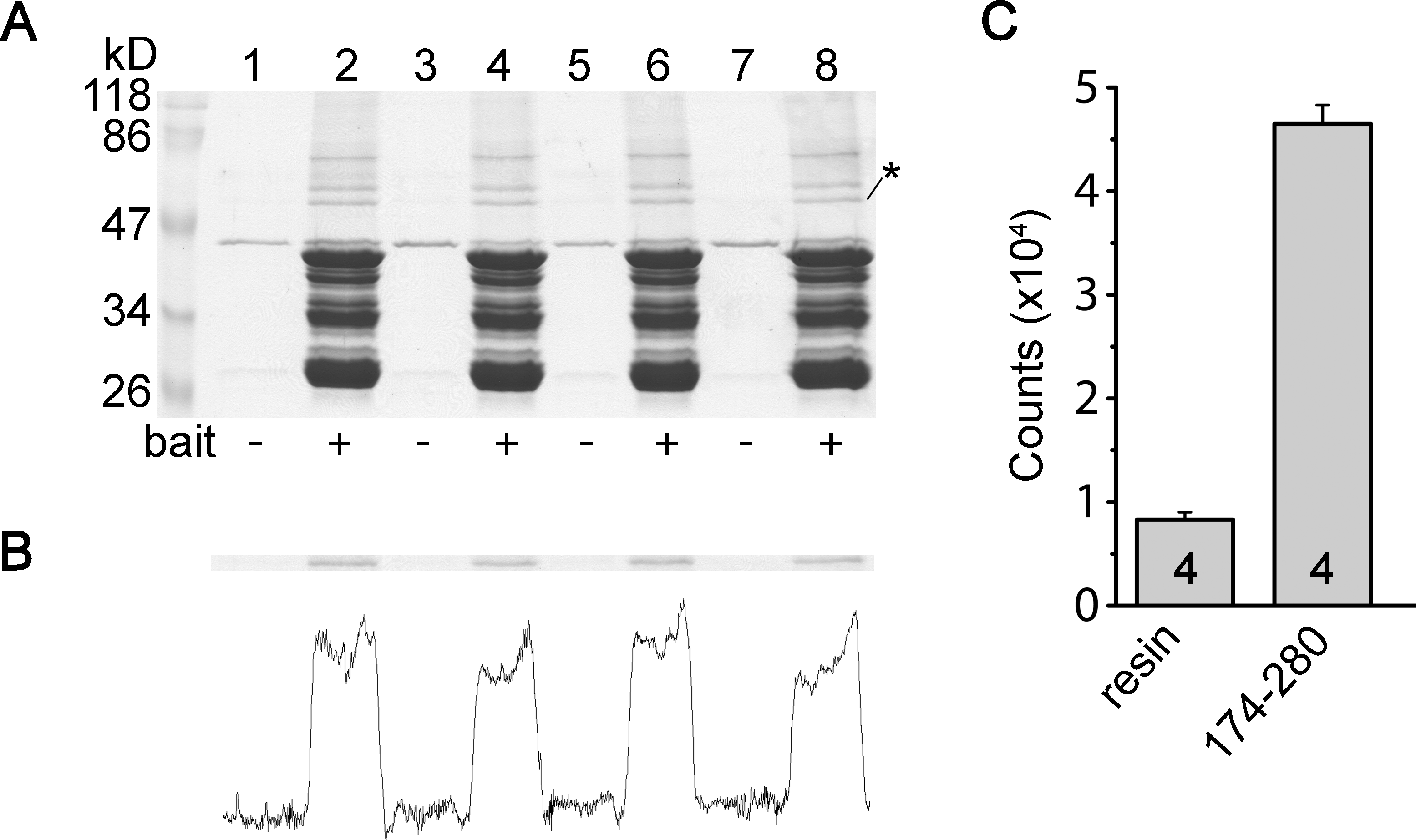

Supplement: Figure S3 — Statistical evaluation of the binding of tubulin to the cytoplasmic loop of TRESK by Coomassie Blue staining and densitometry. A. Four pairs of independent pull-down assays were performed from mouse brain cytosol with the GST fusion protein containing residues 174–280 of human TRESK (even lanes) or with glutathione agarose (odd lanes). The proteins were separated by SDS-PAGE and stained with Coomassie Brilliant Blue. Tubulin is indicated with an asterisk. B. Tubulin bands were analyzed by densitometry. The densitometry curve from ImageJ software is illustrated under the bands. C. The counts calculated from the densitometry curve without background subtraction are shown as a column diagram with error bars (S.E.) for the control resin and fragment 174–280 reactions. Significantly higher amount of tubulin interacted with the bait protein than with the control glutathione agarose (p<10−5, Student's t-test). In the bars, sample numbers are shown. (TIF) [file pone.0097854.s003.tif]

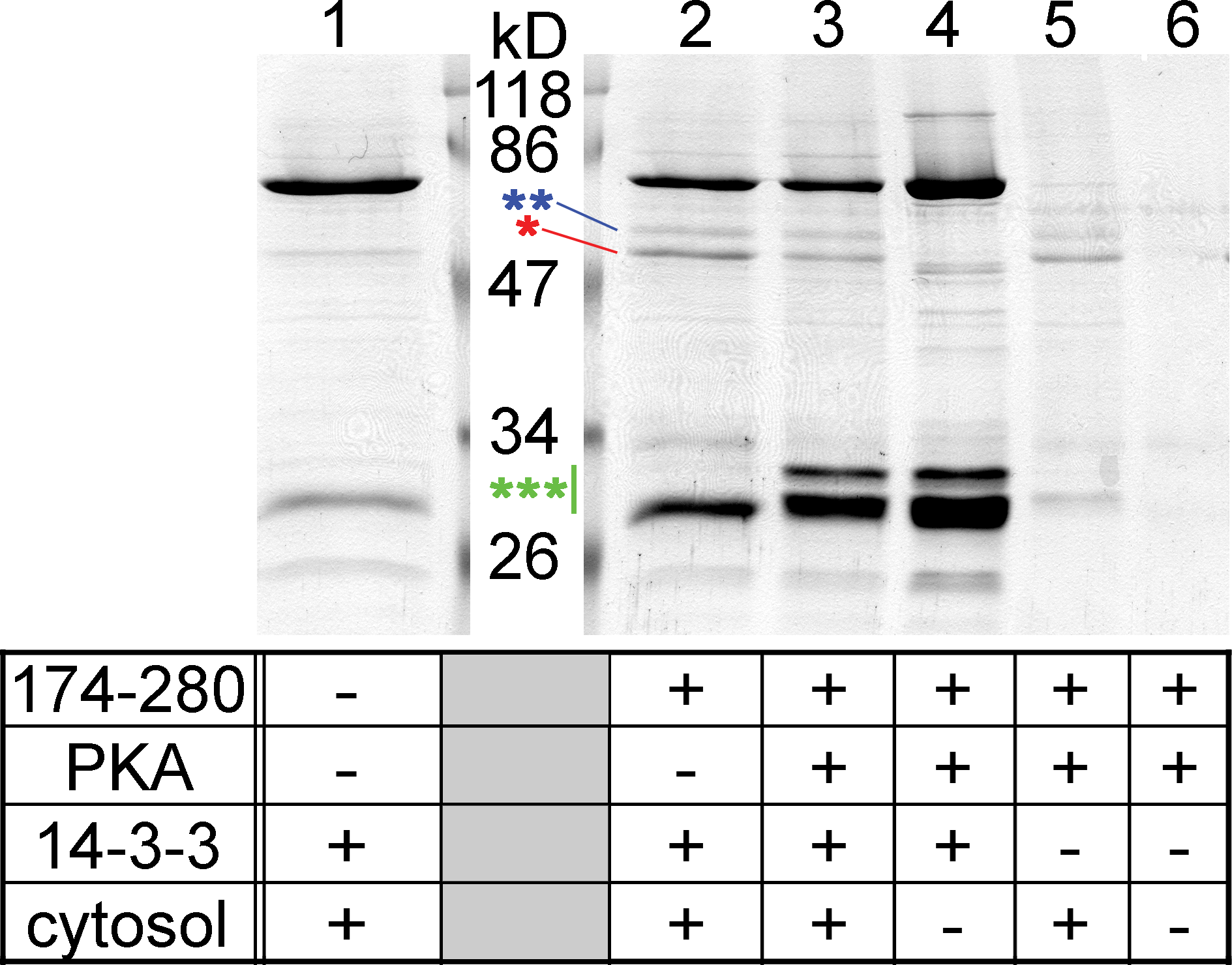

Supplement: Figure S4 — The experiment in Fig. 8 was reproduced. The experiment shown in Fig. 8 was repeated, and practically the same result was obtained. Note that the band in the 30 kD range (indicated with a green triple asterisk) in lane 2 was more intense than the nonspecific band in the Ni-NTA control reaction (lane 1) in this experiment. This suggests that a low amount of 14-3-3 bound to the bait even if it had not been phosphorylated with PKA in advance. The presence of protein kinases and ATP in the brain cytosol may explain the limited phosphorylation of TRESK-loop-His8 and the binding of 14-3-3 in this case. The bait proteins (and the 19 kD marker band) were allowed to run out of the gel during electrophoresis in this experiment to obtain higher resolution in the 50 kD range. (TIF) [file pone.0097854.s004.tif]
